# Sedentary Behavior and Problematic Smartphone Use in Chinese Adolescents: The Moderating Role of Self-Control

**DOI:** 10.3389/fpsyg.2019.03032

**Published:** 2020-01-21

**Authors:** Ming-Qiang Xiang, Long Lin, Zi-Rong Wang, Jin Li, Zebo Xu, Min Hu

**Affiliations:** ^1^Department of Sports and Health, Guangzhou Sport University, Guangzhou, China; ^2^Department of Graduation, Guangzhou Sport University, Guangzhou, China; ^3^Department of Linguistics and Modern Languages, The Chinese University of Hong Kong, Sha Tin, China

**Keywords:** sedentary behavior, problematic smartphone use, self-control, adolescents, exercise interventions

## Abstract

This study investigated smartphone use characteristics including the purpose of smartphone use (i.e., leisure, learning, or work) and situational smartphone use (i.e., sitting, standing, or moving about) in Chinese adolescents. Moreover, it tested the moderating role of self-control in the link between sedentary behavior and problematic smartphone use. A total of 947 adolescents completed measures of the purpose of their smartphone use, situational smartphone use, sedentary behavior, self-control, time on smartphone, and smartphone addiction. Results showed that the majority of smartphone use was for leisure and learning, and 90.9% of adolescents reported typically sitting as they used the smartphone. Problematic smartphone use was positively correlated with sedentary behavior and negatively correlated with self-control. Moreover, the relationship between sedentary behavior and problematic smartphone use was moderated by self-control, in that the negative correlation was stronger for adolescents with low self-control and weaker for those with high self-control. These results contribute to the understanding of when sedentary behavior is associated with problematic smartphone use. Several limitations and implications are discussed in this study.

## Introduction

With the development of internet-based smart devices, the prevalence of smartphone use has steadily increased worldwide, including in China. The Ministry of Industry and Information Technology of China (2019) announced that more than 1.57 billion Chinese people had their own mobile phones in 2018. Although the smartphone brings conveniences to people’s digital lifestyle, many problematic smartphone usages have also emerged among younger people, including in Chinese adolescents (Liu et al., 2017, 2018a). With its special features of convenience, immediacy, and affordability, today’s smartphone allows people to call, receive and send messages, surf the internet, play mobile games, and update social networking sites (e.g., Facebook and WeChat) almost anywhere and anytime. Historically, these activities were defined as sedentary behaviors (Rosenberg et al., 2010). More importantly, a consistent body of literature showed that more than 80% of people reported typically sitting when using their device (Barkley and Lepp, 2016; Long et al., 2016; Lee et al., 2017; Fennell et al., 2019), and such inactive behaviors are linked to numerous comorbidities including obesity, cardiovascular disease, and metabolic syndrome (Owen et al., 2010). Due to the prevalence of smartphone usage and the access it provides to sedentary behaviors, it is important to expand our understanding of the behavioral health implications potentially related to smartphone use. This study considers the relationship between sedentary behavior and problematic smartphone use and tests the moderating role of self-control in the relationship between those variables.

### Sedentary Behavior and Problematic Smartphone Use

An excessive amount of sedentary behavior in adolescents is a growing problem in China (Lu et al., 2017). Although many factors may affect sedentary behavior, the association of smartphone use with sedentary behavior and leisure time is well demonstrated, much as sedentary behavior has been linked with traditional forms of screen-based activities (e.g., watching television, playing video games, and surfing the internet). For example, 70% of college students and 81% of adults reported using their smartphone for leisure purposes (Barkley and Lepp, 2016; Fennell et al., 2019). Prior research has also found that sedentary behaviors are strong predictors of smartphone usage time in college students as well as adults aged 18–80, indicating that excessive smartphone use may increase sedentary behaviors and distract from physical activity (Barkley and Lepp, 2016; Fennell et al., 2019). Furthermore, after controlling for other factors linked to physical quality (e.g., gender, percentage of body fat, and self-efficacy for exercise), excessive smartphone use can ultimately result in reduced cardiorespiratory fitness levels among college students (Lepp et al., 2013).

Prior studies have focused mainly on college students or adults. However, little is known about the relationship between sedentary behavior and problematic smartphone use in adolescents. Adolescence represents a critical transitional stage of development, during which personal lifestyle choices and behavior patterns are established. Clearly, more studies are required to explore smartphone use characteristics as well as the relationship between sedentary behavior and problematic smartphone use in adolescents.

### Self-Control as a Moderator

Self-control, defined as the ability to volitionally control or override inner desires and external temptations in order to achieve long-term goals (Tangney et al., 2004), is an important dispositional trait for generating adaptive personal and social responses. High self-control is positively associated with desirable life outcomes, including better physical and mental health, higher academic performance, and more wealth (Tangney et al., 2004; Moffitt et al., 2011). In contrast, a deficit in self-control is positively associated with undesirable outcomes or social problems, such as binge eating, aggression, depression, and addiction (Denson et al., 2011; Özdemir et al., 2014; Pearson et al., 2018). Problematic smartphone use is generally described as an addictive behavior or incapacity to control cravings to use smartphones (Walsh et al., 2010; Liu et al., 2018b). According to self-regulation theory, addictive behaviors primarily result from failures of self-regulation. Poor self-control might limit an individual’s ability to reduce cravings and restrain addiction (Köpetz et al., 2013; Gökçearslan et al., 2016). This lack of self-control is intrinsically linked to problematic smartphone use. Indeed, a consistent body of research has shown that low levels of self-control not only predict high-frequency usage of smartphones (Wilmer and Chein, 2016; Berger et al., 2018) but also link to smartphone addiction such as withdrawal symptoms, mood changes and cyberspace-oriented relationship (Gökçearslan et al., 2016; Jiang and Zhao, 2016; Yun et al., 2016; Berger et al., 2018).

Self-control is also correlated with sedentary behavior. For example, preliminary evidence showed that lower inhibition-control was directly or indirectly associated with sedentary behavior (Hoang et al., 2013). In modern life, although individuals often plan and intend to exercise, they do not always transform their intentions into actual exercise behavior. According to behavioral economics theory, sedentary behavior can be perceived as an easy, “low-cost” activity with immediate reinforcements, such as fun and entertainment, whereas physical activity can be viewed as a “high-cost” commitment, requiring effort and few immediate reinforcements (Epstein, 1998). Thus, Martin Ginis and Bray (2010) suggested that the capacity to block out sedentary behavior and promote physical activity requires self-control.

With in-depth study, self-control not only negatively correlated with personal and social problems, but also played an important, protective moderator role in the relationship between negative factors and their outcomes. Cooper et al. (2017) found that self-control could buffer the correlation between school burnout and emotional dysregulation. Furthermore, Liu et al. (2018b) found that the direct association between mindfulness and poor sleep quality and the indirect association through rumination were both moderated by self-control among adolescents. These two associations are stronger for those with low self-control and weaker for those with high self-control.

To the best of our knowledge, it remains unclear how sedentary behavior and self-control interact to affect problematic smartphone use. To fill these gaps, it is worth constructing a moderation model to test the moderating variable of self-control in the association between sedentary behavior and problematic smartphone use. The moderation model would contribute to understanding of how self-control protects individuals from problematic smartphone use.

### Hypotheses

This study aims to investigate smartphone use characteristics and explore the relationship between sedentary behavior, self-control and problematic smartphone use in Chinese adolescents. Specifically, we hypothesized the following in a sample of adolescents.

**Hypothesis 1**: Because a smartphone provides a variety of leisure (e.g., videos and game) and learning (e.g., English materials) applications, the majority of smartphone use will be for leisure and learning purposes in Chinese adolescents.
**Hypothesis 2**: Because the smartphone makes it easier to access traditionally sedentary and screen-based activities, smartphone use will occur primarily while sitting.
**Hypothesis 3**: Because smartphone use primarily occurs while sitting, the sedentary behavior will be positively related to problematic smartphone behaviors.
**Hypothesis 4**: Because prior researches have indicated that self-control plays an important protective role, the relationship between sedentary behaviors and problematic smartphone use was moderated by self-control.

## Materials and Methods

### Participants and Data Collection

We used a descriptive transversal design study which was approved by the Human Experimental Ethics Board of Author’s University (Reference number: 2018LCLL-007). With a convenient sampling method, we recruited students from two junior high schools (grade 7 to grade 9) and two senior high schools (grade 10 to grade 12) in the Guangdong province in southern China. In each target school, we randomly chose two or three classes in each grade. Prior to investigation, the parents or guardians of participants were well-informed and their written consent was obtained. A total of 969 Chinese target students were invited to voluntarily participate in the anonymous paper-and-pencil questionnaires survey, which was conducted in classrooms by well-trained college students. All participants completed our survey, but 22 participants were excluded because of missing data on the main variables. Overall, 947 adolescents in the sample were employed, the mean age was 14.13 (SD = 1.79) ranging from 11 to 18 years. There were 489 male students with an average age of 14.13 (SD = 1.71) and 458 female students with an average age of 14.12 (SD = 1.89).

### Measurements

#### Smartphone Use Characteristics

The study evaluated basic demographics (e.g., age, gender, grade, and smartphone ownership), purpose of smartphone use, and situational smartphone use. Regarding purpose of smartphone use, participants were asked to indicate “what percentage of the time the smartphone is used for the following purposes: leisure, learning, work.” The list of items was designed to ensure that the sum of the three responses totaled 100% (Lepp et al., 2014). The situational smartphone use was assessed with three fixed choice items: “When I am using my smartphone, I am most often: (a) sitting, (b) standing, or (c) moving about.”

#### Sedentary Behavior

Sedentary behavior (i.e., sitting) was assessed with two items from the International Physical Activity Questionnaire (IPAQ) (Craig et al., 2003; Bauman et al., 2009). Participants reported the average number of minutes of each week day (or each weekend day) they spent sitting. Weekly sedentary behavior was calculated using the following equation: weekly sedentary behavior = [(5 × minutes of sitting per week day) + (2 × minutes of sitting per weekend day)]/7.

#### Self-Control

We used the China short form of the trait self-control scale (SCS) (Tan, 2008) revised from the original version by Tangney et al. (2004), including a scale of 19 items. The SCS measures five aspects of self-control abilities:(1) deliberate and non-impulsive action, (2) healthy habits, (3) resistance to temptation, (4) work ethic, and (5) moderation in seeking diversions. Participants assessed each item on a five-point scale from 1 (not at all like me) to 5 (very much like me). Higher scores on this scale indicate a stronger capability for self-control.

#### Problematic Smartphone Use

Two questionnaires were selected to assess the problematic smartphone use, including time on smartphone use and smartphone addiction scale.

Time on smartphone was assessed with two items using a method followed by Lepp et al. (2014). Participants were asked to estimate their average time spent (in minutes) on their smartphone for each weekday and each weekend day. This self-report measure is associated with objectivity and other self-reported measures of smartphone use, which were applied in previous studies (Barkley and Lepp, 2016; Fennell et al., 2019). Weekly smartphone use was calculated using the following equation: weekly smartphone use = [(5 × minutes of smartphone use per week day) + (2 × minutes of smartphone use per weekend day)]/7.

Smartphone addiction was assessed by the ten-item Smartphone Addiction Scale-Short Version (SAC-SV) for adolescents (Kwon et al., 2013). The scale was translated by independent researchers using the parallel translation method. Any disagreement was resolved by discussion or, if required, by consulting a third author. Participants assessed each item on a six-point scale: 1 (fully disagree) to 6 (fully agree). The total scores ranged from 10 to 60. Kwon et al. (2013) suggested cut-off points per gender (boys 31 and girls 33) to classify the smartphone addiction group (SAG) or non-smartphone addiction group (non-SAG).

### Statistical Analysis

All data analysis was performed using SPSS 23.0. A *p*-value of 0.05 indicated statistical significance. We first computed descriptive statistics for the whole sample, and then compared differences between SAG and non-SAG with continuous variables using independent *t*-test and categorical variables using χ^2^. Additionally, we used a Pearson correlation analysis to assess the association between sedentary behavior, physical activity, self-control, smartphone use, and smartphone addiction. Finally, we performed moderation analyses using Hayes (2013) bootstrapping Process for SPSS (Model 1) to examine whether the sedentary behavior effect on time of smartphone use and smartphone addiction were moderated by self-control. All continuous variables were standardized and the interaction terms were computed based on standardized scores. The bootstrapping method produced 95% bias-corrected confidence intervals of these effects from 5000 resamples of the data (Hayes, 2013).

## Results

### Descriptive Statistics and Comparative Analysis

Based on of results of previous studies (Kwon et al., 2013; Lopez-Fernandez, 2017), the scoring 32 was selected as the cut-off to identify smartphone addiction because there was no significant difference between gender in SAS-SV scores (*t* = 0.69, *p* = 0.49). Table 1 presented the socio-demographic and smartphone use characteristics between those with and without SA.

**TABLE 1:** Comparisons of variables between subjects with and without SA.

	All (*n* = 947)	Non-SAG (*n* = 776)	SAG (*n* = 171)
Age (years)	14.13 ± 1.79	14.05 ± 1.84	14.47 ± 1.55^∗∗^
**Gender**			
Boys	489 (51.6%)	400 (81.8%)	89 (18.2%)
Girls	458 (48.4%)	376 (82.1%)	82 (17.9)
Ratio of smartphone ownership	698 (73.7%)	557 (71.8%)	141 (82.5%)^∗∗^
**Purpose of smartphone use (%)**			
Leisure	43.87 ± 25.65	40.96 ± 24.48	57.06 ± 26.78^*⁣**^
Learning	45.94 ± 24.81	48.85 ± 24.20	32.75 ± 23.28^*⁣**^
Work	9.59 ± 13.56	9.54 ± 13.41	9.82 ± 14.27
**Situational of smartphone use**			
Sitting	861 (90.9%)	711 (91.6%)	150 (87.7%)
Standing	24 (2.5%)	20 (2.6%)	4 (2.3%)
Moving about	62 (6.5%)	45 (5.8%)	17 (9.9%)
**Sedentary behavior (min/day)**			
Weekday	465.99 ± 126.89	461.52 ± 125.12	485.76 ± 133.01^∗^
Weekend	382.32 ± 143.82	371.57 ± 140.08	424.67 ± 149.82^*⁣**^
Total	442.08 ± 112.74	436.31 ± 110.40	468.30 ± 119.68^∗∗^
Self-control	3.59 ± 0.53	3.68 ± 0.51	3.21 ± 0.43^*⁣**^
**Smartphone use (min/day)**			
Weekday	13.95 ± 33.15	12.58 ± 30.47	20.15 ± 42.84^∗^
Weekend	171.84 ± 158.16	150.85 ± 141.52	267.09 ± 191.69^*⁣**^
Total	59.06 ± 54.61	52.09 ± 48.47	90.70 ± 68.25^*⁣**^
Smartphone addiction	25.09 ± 7.44	22.59 ± 5.35	36.46 ± 4.39^*⁣**^

*SAG, smartphone addiction group; Non-SAG, non-smartphone addiction group. ^∗^*p* < 0.05, ^∗∗^*p* < 0.01, ^∗∗∗^*p* < 0.001.*

A total of 947 adolescent subjects participated in this study; 698 of the participants (73.7%) owned a smartphone with 776 of them in the non-SAG (81.9%) and 171 in the SAG (18.1%). When these two groups were compared, there were no significant differences in their genders; however, the age was significantly greater in the SAG (*t* = 2.76, *p* = 0.006), and the proportion of smartphone ownership was also significantly higher in the SAG (χ^2^ = 8.24; *p* = 0.004).

Regarding purpose of smartphone use, on average, participants categorized 43.87% of their smartphone use as leisure, 45.94% as learning, and 9.59% as work. On close inspection, the SAG had significantly greater smartphone use as leisure (*t* = 7.92, *p* < 0.001), but lower smartphone use as learning (*t* = -7.65, *p* < 0.001) compared with the non-SAG. In this sample, 90.9, 2.5, and 6.5% of participants reported that they are most likely sitting down, standing and moving, respectively, while using their smartphone. However, there was no significant difference in usage preferences between SAG and non-SAG regarding these three postures (χ^2^ = 0.014, *p* = 0.906).

The mean daily sitting was 442.08 (SD = 112.74) min/day. The mean self-control score was 3.59 (SD = 0.53) units. When these two groups were compared, the SAG showed significantly higher sedentary behavior on weekdays (*t* = 2.26, *p* = 0.024) and weekends (*t* = 4.43, *p* < 0.001), as well as higher overall minutes being sedentary (*t* = 3.38, *p* < 0.001). They also showed significantly lower self-control than the non-SAG (*t* = 11.17, *p* < 0.001).

Regarding daily smartphone use, mean smartphone use time in weekdays, weekends, and total minutes was 13.95 (SD = 33.15), 171.84 (SD = 158.16), and 59.06 (SD = 54.61) min/day, respectively. The SAG had significantly greater smartphone use weekdays (*t* = 2.71; *p* = 0.007), weekends (*t* = 9.07; *p* < 0.001), and for total minutes of use (*t* = 8.69; *p* = 0.005).

### Correlation Analyses

The descriptive statistics and correlation matrix are presented in Table 2. Sedentary behavior was positively correlated with time on smartphone use (*p* < 0.001) and smartphone addiction (*p* < 0.001), and negatively correlated with self-control (*p* < 0.05). Self-control was negatively associated with time on smartphone use (*p* < 0.001) and smartphone addiction (*p* < 0.001).

**TABLE 2:** Descriptive statistics and correlations between variables.

Variables	M	SD	1	2	3
1. Sedentary behavior	442.08	112.74			
2. Self-control	3.59	0.53	−0.07^∗^		
3. Time on smartphone use	59.06	54.61	0.23^∗∗∗^	–0.23^∗∗∗^	
4. Smartphone addiction	25.09	7.44	0.12^∗∗∗^	–0.54^∗∗∗^	0.34^∗∗∗^

**N* = 947. ^∗^*p* < 0.05, ^∗∗∗^*p* < 0.001.*

### Testing for the Moderation Model

**FIGURE 1:**
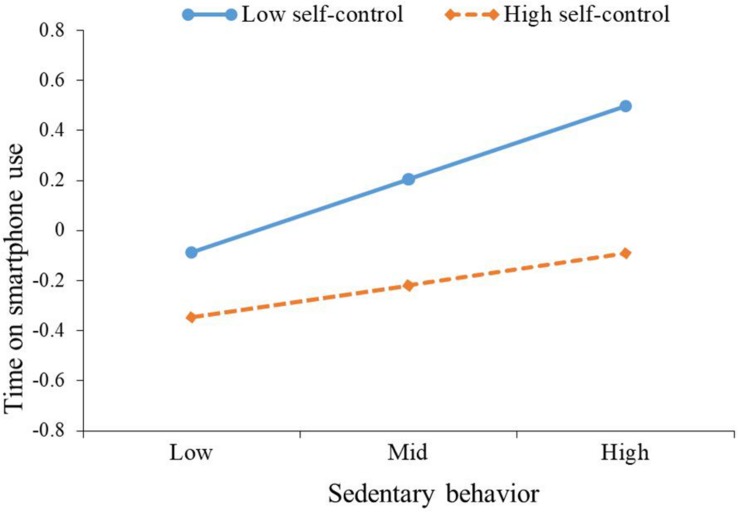
The plot of the relationship between sedentary behavior and time on smartphone use at two levels of self-control.

The main results of moderation analysis generated by Hayes (2013) SPSS macro PROCESS are presented in Table 3. Regarding time on smartphone use, after controlling for gender and age, sedentary behavior was positively correlated with time on smartphone use (β = 0.15, *p* < 0.001); self-control was negatively correlated with time on smartphone use (β = −0.18, *p* < 0.001); and the interaction of sedentary behavior and self-control was negatively correlated with time on smartphone use (β = −0.08, *p* < 0.01). Namely, self-control moderated the association between sedentary behavior and time on smartphone use. To better understand the moderating effect of self-control, the plot of the relation between sedentary behavior and time on smartphone use at two levels of self-control (1 SD below the mean and 1 SD above the mean) was described in Figure 1. As can be seen from Figure 1 and the conditional effects analysis in Table 4, for individuals with low self-control (1 SD below the mean), sedentary behavior was positively associated with time on smartphone use (β = 0.23, *p* < 0.001), while this association (β = 0.07, *p* > 0.05) was not significant for individuals with high self-control (1 SD above the mean).

**TABLE 3:** Moderation analysis.

Outcomes	Predictors	β	*t*	LLCI	ULCI
Time on smartphone use	Gender	–0.07	–1.22	–0.19	0.04
	Age	0.24	7.50^∗∗∗^	0.17	0.30
	Sedentary behavior	0.15	4.85^∗∗∗^	0.09	0.21
	Self-control	–0.18	–6.04^∗∗∗^	–0.24	–0.12
	Sedentary behavior × self-control	–0.08	–2.66^∗∗^	–0.13	–0.02
Smartphone addiction	Gender	0.01	0.18	–0.10	0.12
	Age	0.04	1.45	–0.01	0.10
	Sedentary behavior	0.06	2.17^∗^	0.01	0.12
	Self-control	–0.53	–19.33^∗∗∗^	–0.59	–0.48
	Sedentary behavior × self-control	–0.06	−2.23^∗^	–0.11	–0.01

**n* = 947. Bootstrap sample size = 5000. CI, confidence interval; LL, low limit; UL, upper limit. ^∗^*p* < 0.05, ^∗∗^*p* < 0.01, ^∗∗∗^*p* < 0.001.*

**TABLE 4:** Conditional effects results at values of moderators.

Outcomes	Predictor	Values of self-control	β	*t*	LLCI	ULCI
Time on smartphone use	Sedentary behavior	Mean - SD	0.23	5.47^∗∗∗^	0.15	0.31
		Mean	0.15	4.85^∗∗∗^	0.09	0.21
		Mean + SD	0.07	1.69	–0.01	0.16
Smartphone addiction	Sedentary behavior	Mean - SD	0.12	3.17^∗∗^	0.05	0.19
		Mean	0.06	2.17^∗^	0.01	0.12
		Mean + SD	0.01	0.06	–0.08	0.08

**n* = 947. Bootstrap sample size = 5000. CI, confidence interval; LL, low limit; UL, upper limit. ^∗^*p* < 0.05, ^∗∗^*p* < 0.01, ^∗∗∗^*p* < 0.001.*

**FIGURE 2:**
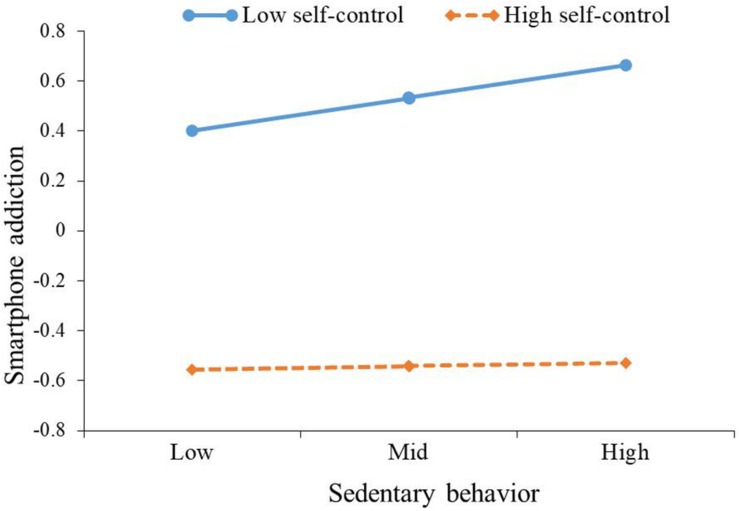
The plot of the relationship between sedentary behavior and smartphone addiction at two levels of self-control.

As can be seen from the moderation model for predicting smartphone addiction, after controlling for gender and age, sedentary behavior was positively correlated with smartphone addiction (β = 0.06, *p* < 0.05), while the interaction of sedentary behavior and self-control was negatively correlated with smartphone addiction (β = −0.06, *p* < 0.05). In other words, self-control moderated the association between sedentary behavior and smartphone addiction. The plot of the relation between sedentary behavior and smartphone addiction at two levels of self-control (1 SD below the mean and 1 SD above the mean) was described in Figure 2. As can be seen from Figure 2 and the conditional effects analysis in Table 4, for individuals with low self-control (1 SD below the mean), sedentary behavior was positively associated with smartphone addiction (β = 0.12, *p* < 0.01), while this association (β = 0.01, *p* > 0.05) was not significant for individuals with high self-control (1 SD above the mean).

## Discussion

A descriptive transversal design was carried out to investigate smartphone use characteristics in this study, including the purpose of smartphone use (i.e., leisure, learning, or work) and situational smartphone use (i.e., sitting, standing, or moving about). Furthermore, this study examined the moderating role of self-control between sedentary behavior and problematic smartphone use in Chinese adolescents. In the current study, the proportion of adolescents who own a smartphone is 73.7% among Chinese adolescents. They spend more time on their smartphone on weekends because of the heavy learning tasks and explicit prohibition in school on smartphone use at weekday (Gao et al., 2014). The prevalence of potential smartphone addiction was 18.1%, similar to the range reported in the Kwon et al. (2013) for Korean adolescents (16.6% in boys, 26.6% in girls).

In this study, participants reported that, on average, 43.87% of their smartphone use was for leisure, 45.94% for learning, and 9.59% for work, indicating the percentage of total daily smartphone use devoted to leisure and learning was similar in adolescents. Thus, Hypothesis 1 was supported. These results are inconsistent with previous studies that have shown the majority of smartphone use was for leisure in college students (70–88% use for leisure) (Lepp et al., 2013, 2017; Barkley and Lepp, 2016) and adults (61% use for leisure) (Fennell et al., 2019). However, the purpose of smartphone use was significantly different between SAG and non-SAG, namely, the SAG had greater smartphone use for leisure, while the non-SAG had greater smartphone use for learning. These results combined with previous logistic regression analysis results showed that levels of smartphone addiction were reduced when smartphones were used for learning (Lee et al., 2017), suggesting that parents and teachers should provide guidance for adolescents about specific functions of smartphone use, such as learning or searching for information to reduce smartphone addiction.

Regarding situational use, 90.9% of all adolescents reported using the smartphone primarily while sitting. This is very similar to previous research in samples of college students (87%) and adults (81%) (Barkley and Lepp, 2016; Fennell et al., 2019). It seems that smartphones, despite their portability and mobility, are primarily sedentary devices for all individuals regardless of age. So Hypothesis 2 was verified.

Congruent with previous studies (Barkley and Lepp, 2016; Fennell et al., 2019), our finding showed that sedentary behavior was negatively correlated with use time on smartphones, suggesting that individuals who allocated more time for daily sitting use smartphones for greater periods. But beyond the time on smartphone use of previous studies, our results demonstrated that sedentary behavior was also negatively associated with smartphone addiction, indicating that our findings more comprehensively revealed the relationships between sedentary behavior and problematic smartphone use. Thus, Hypothesis 3 was supported. In addition, prior studies have identified sedentary behavior as an independent risk factor for cardiovascular disease (Katzmarzyk et al., 2009; Carter et al., 2017), which is worrisome as individuals with smartphone addiction spent more time on sedentary behavior and are at greater risk for cardiovascular disease than those without smartphone addiction.

Unlike sedentary behavior, the results of relationship between physical activity and problematic smartphone use were inconsistent in prior studies. For example, some researchers found there was no direct relationship between volume of daily physical activity and time on smartphone use (Barkley and Lepp, 2016; Fennell et al., 2019), while Kim et al. (2015) revealed that average number of walking steps per day negatively correlated with smartphone addiction. Other researches demonstrated that using the smartphone for texting during treadmill exercise may reduce participation in vigorous intensity exercise (Rebold et al., 2016), while using the smartphone for listening to music has been shown to increase exercise intensity (Rebold et al., 2015), suggesting the relationship between physical activity and smartphone depending on the aspect of smartphone functions. Based on the results from previous studies, we speculate that smartphone use may increase sedentary behavior by using traditional forms of screen-based apps while simultaneously prompt physical activity by using health related apps.

Novel to our study was our demonstration that not only the relationships between sedentary behavior and time spent on smartphone use but also between sedentary behavior and smartphone addiction were moderated by self-control. These two associations were stronger for individuals with low self-control than for those with high self-control. Therefore, Hypothesis 4 was verified, which indicates the sense of moderation of self-control in the relationship between sedentary behavior and problematic smartphone use. These findings are consistent with recent theorizing on the trait of self-control (de Ridder et al., 2012; Hofmann et al., 2014b) and prior studies (Cooper et al., 2017; Liu et al., 2018b) indicating the protective role of self-control. Individuals who are high in self-control are more likely to reduce problematic smartphone use even though their sedentary behavior is at a high level. Presumably, these individuals have developed a good coping strategy and self-control capacities that help them to avoid using the smartphone when they are sitting. In contrast, smartphone use among persons who are low in self-control seems to be more strongly influenced by sedentary behavior. An explanation could be that these individuals’ attention was generally hijacked by the smartphone, which leads individuals to respond immediately to smartphone signals when they are sitting (Berger et al., 2018).

## Limitations and Implications

Several limitations of the present study are noteworthy. First, due to the cross-sectional survey design in this study, causal relationships between sedentary behavior and problematic smartphone use should be interpreted with great caution. Future research may adopt longitudinal or experimental study models to strictly identify the causal relationships among these variables. Second, due to social desirability and other biases, the self-report method might inflate shared method variance and restrict the validity of the data. Future research using objective methods (such as ActiGraph accelerometers and smartphone apps) to assess the sedentary behavior and smartphone use may be necessary to address this. Third, self-control can be subdivided into trait self-control and state self-control; however, only the trait self-control was considered in our study, potentially limiting the utilization of the present study. Future research should try to investigate both state and trait self-control.

Despite the above limitations, the results of this study contribute to an expanding of the scope of interventions geared toward preventing problematic smartphone use in adolescents. Our data show that sedentary behavior was negatively correlated with problematic smartphone use. Although we cannot determine causal relationships between sedentary behavior and problematic smartphone use, reducing sedentary behavior is undoubtedly beneficial for alleviating problematic smartphone use. In fact, China’s government has enacted a “National Teenagers’ Sunny Sports Program” with the goal of having students do 1 h of exercise every day to promote physical activity and reduce sedentary behaviors. We speculate that such a program is not only useful in the field of physical fitness but also in curbing excessive or problematic smartphone use. Other interventions could target adolescents with low self-control by raising their awareness of their tendency to problematic smartphone use and launching evidence-based public health programs for improving self-control levels. Fortunately, promising results have been found in prior research on self-control training (Hofmann et al., 2014a; Friese et al., 2017). One big advantage of such trainings may be the high domain-general capacity; training in self-control in one field may lead to broad improvements in other fields over time. For example, Zou et al. (2016) have found that participating in 5 weeks of aerobic exercise (physical self-control) can increase self-control after ego-depletion in terms of pain tolerance. These pieces of evidence give reason to assume that adolescents low in self-control could benefit from exercise training, leading them to reduced problematic smartphone use.

## Conclusion

This study has found that the majority of smartphone use was for leisure and learning, which was positively associated with sedentary behavior in Chinese adolescents. Furthermore, results of this study provided evidence that self-control exerts a moderating role on the impact of sedentary behavior on adolescents with problematic smartphone use. In other words, strengthening self-control may be effective in helping adolescents with sedentary behavior to limit their problematic smartphone use. The current study expands the pediatric literature on sedentary behavior and problematic smartphone use during the potentially critical developmental period of adolescence and points to the need to launch evidence-based exercise interventions and self-control training for adolescents at risk for problematic smartphone use.

## Data Availability Statement

The datasets generated for this study are available on request to the corresponding author.

## Ethics Statement

The studies involving human participants were reviewed and approved by the Human Experimental Ethics Board of Guangzhou Sports University. The patients/participants provided their written informed consent to participate in this study.

## Author Contributions

M-QX, LL, and MH contributed to the conception and design of the study. Z-RW and JL organized the database. M-QX and LL analyzed the data. M-QX and Z-RW wrote the first draft of the manuscript. MH and ZX contributed to the manuscript revision, read, and approved the submitted version.

## Conflict of Interest

The authors declare that the research was conducted in the absence of any commercial or financial relationships that could be construed as a potential conflict of interest.
